# Risk factors for periorbital dermatitis in patients using dorzolamide/timolol eye drops

**DOI:** 10.1038/s41598-021-97565-0

**Published:** 2021-09-09

**Authors:** Myungjin Kim, Hyoju Jang, Seungsoo Rho

**Affiliations:** 1 Department of Ophthalmology, CHA Bundang Medical Center, CHA University, 59 Yatap-ro, Bundang-gu, Seongnam-si, Gyeonggi-do 463-712 Republic of Korea; 2 SW Bright Eye Clinic, Pocheon, Republic of Korea

**Keywords:** Glaucoma, Eyelid diseases, Adverse effects

## Abstract

This study assessed the clinical risk factors for periorbital dermatitis (PD) after using dorzolamide/timolol eye drops in a total of 1282 glaucoma patients. Both the PD(+) group and the PD(−) group were evaluated using clinical data such as age, sex, dosing duration, presence of benzalkonium chloride (BAK) in the formulation, ocular surgery history (e.g. cataract or glaucoma operations), height, weight, personal history of systemic hypertension, smoking, alcohol consumption, intraocular pressure, best-corrected visual acuity (BCVA), central corneal thickness, axial length, and visual field index (VFI). Univariate analyses showed that shorter dosing duration, higher rate of BAK-included cases, worse BCVA, worse VFI, more systemic hypertension history, and more ocular surgery history were more associated with the PD(+) group than the PD(−) group. The BAK(−) group showed a lower PD rate than the BAK-included group, which was supported by the Kaplan–Meier analysis (log-rank test, p = 0.0014). Multivariate analyses revealed that the probability of PD increased by 8 times if they had a history of ocular surgery and increased by 2.3% when the VFI decreased by 1% (Cox’s hazard regression test, p < 0.001). Therefore, a preservative-free dorzolamide/timolol can benefit the subjects for those who had ocular surgery or who have worse VFI.

## Introduction

Dermatitis in the periorbital area is known to have several causes, such as allergic contact dermatitis, irritant contact dermatitis, and atopic eczema^1^. Allergic contact dermatitis is a delayed-type IV hypersensitivity reaction brought out upon reapplication of an allergen to the skin after previous sensitization. The periorbital skin is susceptible to this reaction due to its extremely thin skin (0.55 mm) compared to other facial areas (− 2 mm), which may allow easier allergen penetration^2^.

Dorzolamide/timolol, one of the most commonly used fixed combination drugs for the management of glaucoma since its first approval in 1998, can also be a causative agent of allergic contact dermatitis. Due to its delayed nature, the onset of symptoms of periorbital dermatitis (PD) after the application of glaucoma eye drops can be overlooked by ophthalmologists. Ever since a case of contact sensitization to timolol eye drops in the periorbital skin and conjunctiva was first reported in 1991^3^, and a case of lid eczema caused by dorzolamide eye drops was first reported in 1998^4^; there was no single clinical report that described the risk factors of PD after using dorzolamide/timolol eye drops. Moreover, it is necessary to determine the effect of benzalkonium chloride (BAK) on PD occurrence since BAK is known as a causative allergen to the skin^5^. We consecutively collected the data of the subjects who were prescribed the preserved or the preservative-free dorzolamide/timolol eye drops to investigate the risk factors of PD and the effect of BAK on PD occurrence.

## Results

A total of 1282 patients were included in the full study population, of which 29 (2.26%) were included in the PD(+) group. More than two-thirds of the patients were diagnosed with primary open-angle glaucoma, and 57.1% (732/1282) were male (Table 1). The mean dosing duration was 879.01 ± 808.43 days and the mean age (years) was 59.81 ± 14.84. There were no statistical differences in age, sex, glaucoma subtype, body mass index (BMI, kg/m^2^), baseline intraocular pressure (IOP), central corneal thickness (CCT), and axial length between the PD(+) and PD(−) groups in the univariate analysis. However, baseline best-corrected visual acuity (BCVA) and visual field index (VFI) were significantly worse (p < 0.001), and the dosing duration was significantly shorter in the PD(+) group than in the PD(−) group (p < 0.001, Table 2). In addition, the proportions of the subjects who used the BAK-included dorzolamide/timolol eye drops, with systemic hypertension history, and with previous ocular surgery history (e.g., cataract, glaucoma surgery) were higher in the PD(+) group than in the PD(−) group (p = 0.032, 0.017, and < 0.001, respectively, Table 2). The worse BCVA and VFI in the PD(+) group were mainly due to the higher proportion of glaucoma operation history compared with the PD(−) group.

**Table 1:** Baseline demographics of total patients with topical dorzolamide-timolol.

Parameter	Number
	(N = 1282)
**Sex**	
Male, n (%)	732 (57.1)
Female, n (%)	550 (42.9)
**Age (years)**	
Mean ± SD	59.81 ± 14.84
Range	9–97
**Diagnosis**	
POAG, n (%)	905 (70.6)
ACG, n (%)	77 (6.0)
NVG, n (%)	94 (7.3)
Others, n (%)	206 (16.1)
**Dosing duration (day)**	
Mean ± SD	879.01 ± 808.43
Range	3–5112
Male, mean ± SD	863.13 ± 796.39
Female, mean ± SD	900.16 ± 824.43
POAG, mean ± SD	977.04 ± 830.71
ACG, mean ± SD	412.39 ± 525.45
NVG, mean ± SD	775.19 ± 705.57
Others, mean ± SD	671.19 ± 736.27

Values are presented as mean ± standard deviation unless otherwise indicated.

*POAG* primary open-angle glaucoma, *ACG* angle-closure glaucoma, *NVG* Neovascular glaucoma.

**Table 2:** Clinical characteristics of study subjects (PD(+) vs PD(−) groups).

	PD(+) (n = 29)		PD(−) (n = 293)		p-value^†^
	Mean	SD	Mean	SD	
Age (years)	65.90	11.32	61.72	13.54	0.109
Male, n (%)	19 (65.5)		153 (52.2)		0.120
Dosing duration (day)	832.86	730.58	1390.89	1090.25	0.001
Diagnosis, POAG, n (%)	24 (82.8)		255 (87.0)		0.463
Diagnosis, ACG, n (%)	2 (6.9)		6 (2.0)		
Diagnosis, NVG, n (%)	1 (3.4)		10 (3.4)		
Diagnosis, others, n (%)	2 (6.9)		22 (7.5)		
BMI (kg/m^2^)	23.97	3.70	23.39	4.70	0.605
Baseline IOP(mmHg)	17.52	6.00	18.10	6.63	0.647
Baseline BCVA (decimal)	0.53	0.39	0.78	0.29	< 0.001
Baseline CCT (μm)	543.81	48.08	546.22	36.91	0.780
Baseline ECC (number)	2406.31	329.01	2462.90	573.39	0.742
Baseline AXL (mm)	24.09	2.68	24.29	1.66	0.590
Baseline VFI (%)	58.26	29.56	85.72	21.02	< 0.001

	N	%	N	%	p-value^‡^
BAK(+)	9	31.0	49	16.7	0.032
HTN	17	58.6	108	36.9	0.017
DM	7	24.1	48	16.4	0.201
Family hx.(Glaucoma)	5	17.2	32	10.9	0.243
Smoking hx	4	13.8	40	13.7	0.783
Coffee	18	62.1	155	52.9	0.924
Alcohol	13	44.8	93	31.7	0.629
Side sleep	7	24.1	53	18.1	0.800
Yoga	0	0.0	12	4.1	0.236
Pipe instrument	0	0.0	1	0.3	0.729
Glaucoma operation hx.	14	48.3	25	8.5	< 0.001
Cataract operation hx.	19	65.5	37	12.6	< 0.001

Values are presented as mean ± standard deviation unless otherwise indicated.

*PD* periorbital dermatitis, *POAG* primary open-angle glaucoma, *ACG* angle-closure glaucoma, *NVG* Neovascular glaucoma.

^†^Independent t-test or chi-square test.

^‡^Chi-square test.

**Figure 1:**
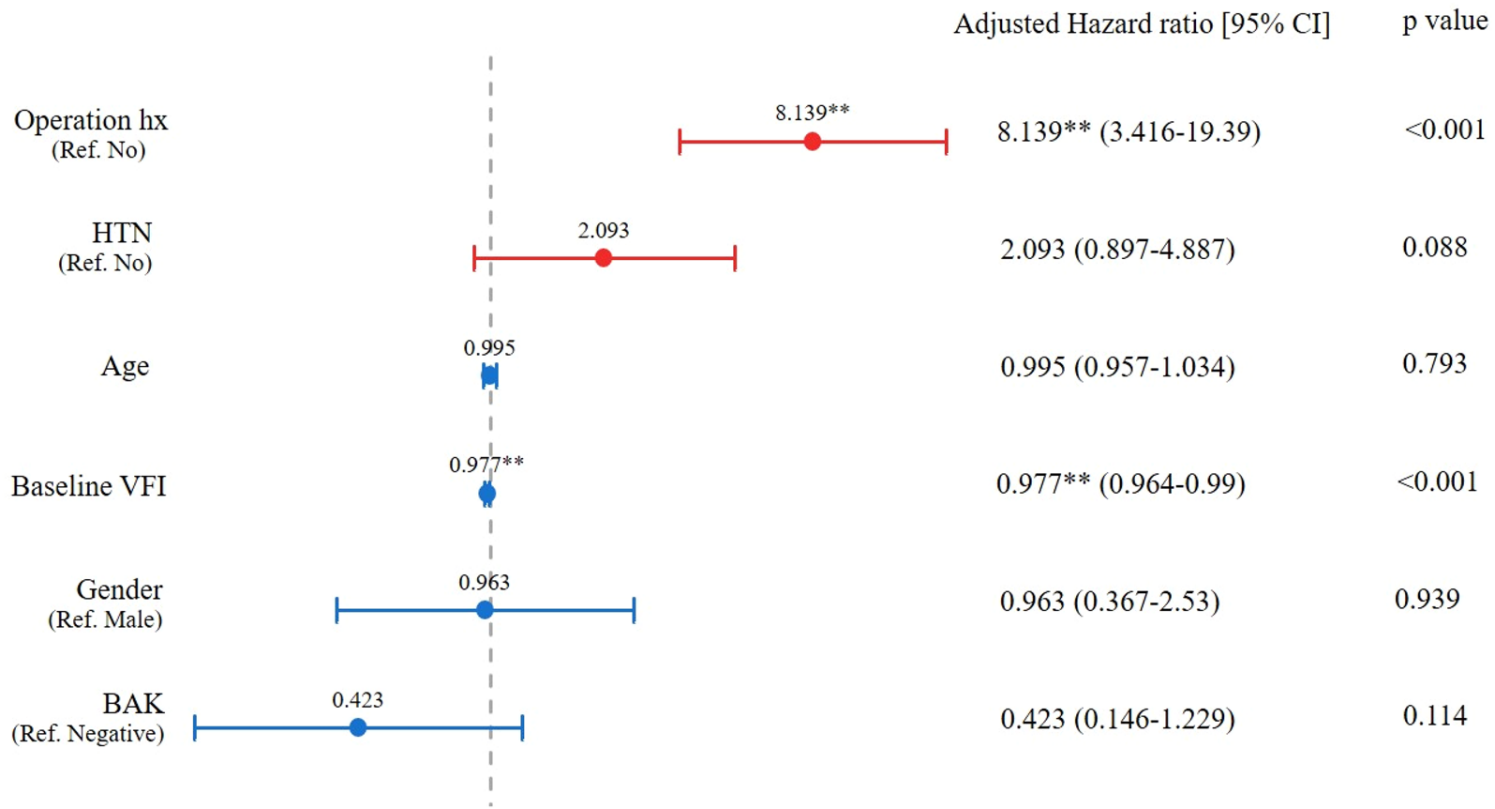
Cox proportional hazard analysis for the risk of periorbital dermatitis with topical dorzolamide-timolol (Operation hx; history of cataract surgery or glaucoma surgery).

**Figure 2:**
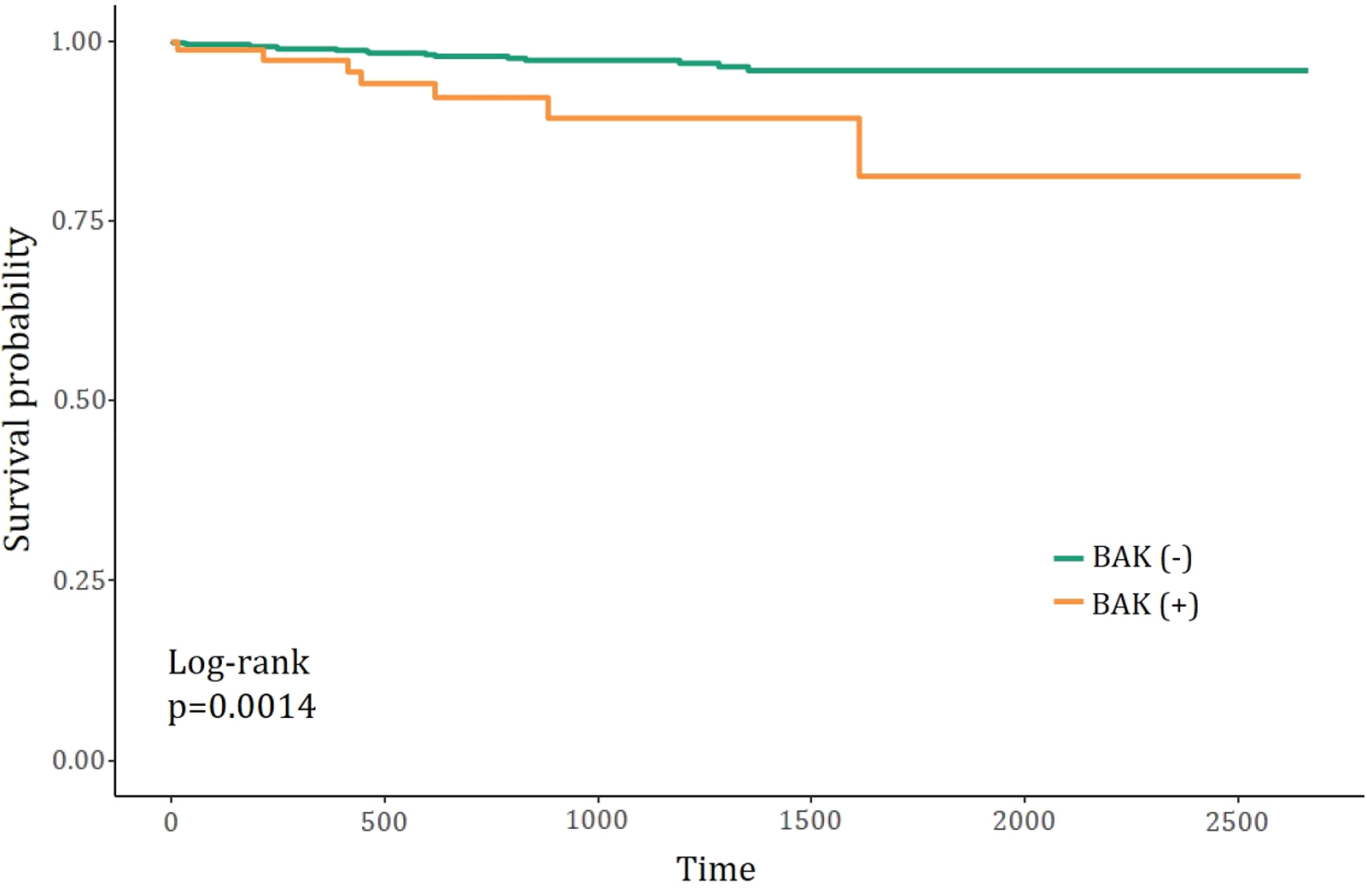
Kaplan-Meyer graph predicting the occurrence of periorbital dermatitis (BAK(+) vs BAK(−) groups).

**Figure 3:**
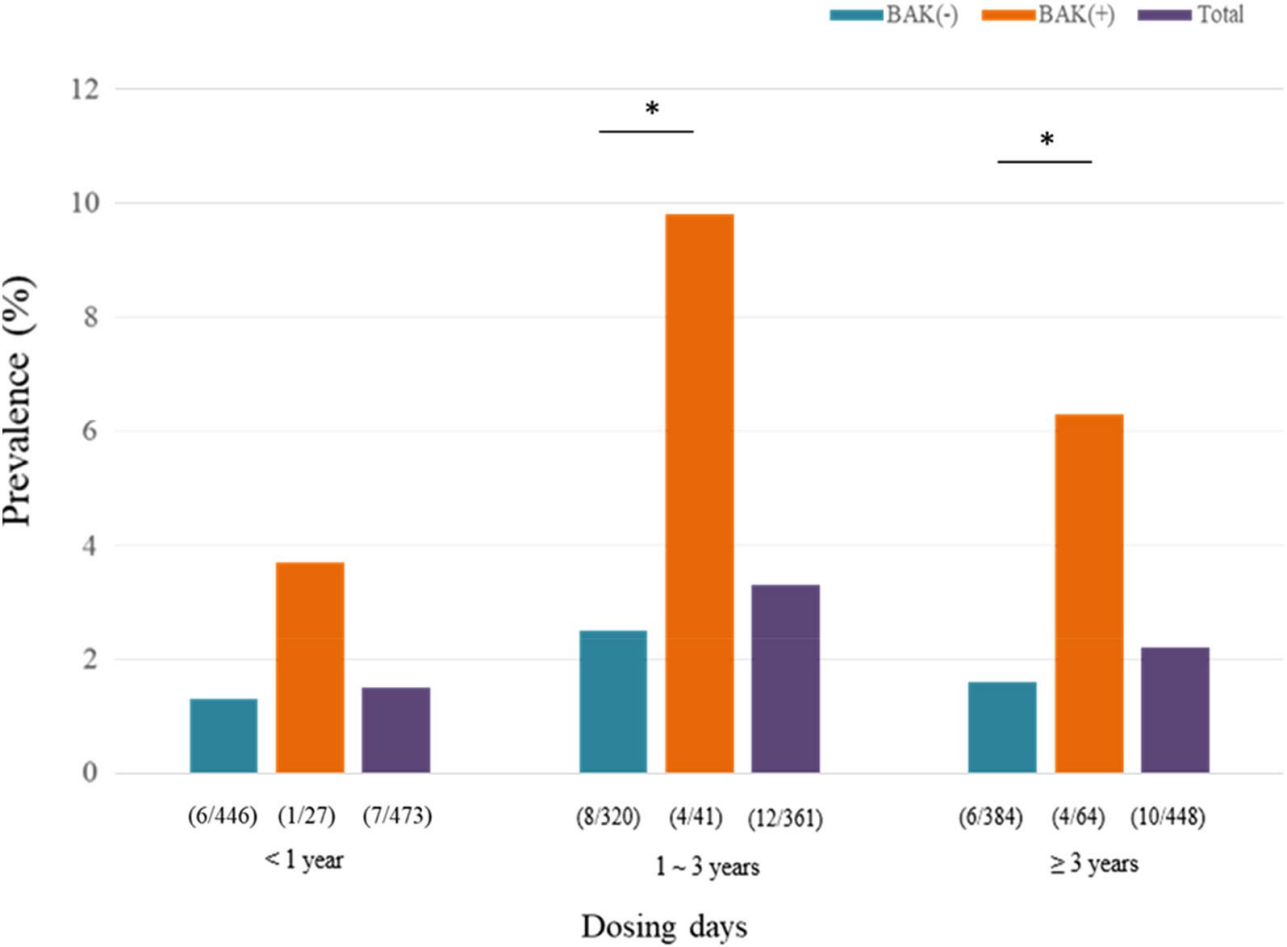
Prevalence of periorbital dermatitis related to dorzolamide/timolol according to dosing days.

In the multivariate analysis using Cox’s hazard regression, ocular surgery history increased the probability of PD by 8.139 times, and the 1% worsening of VFI increased the probability of having PD by 2.3% (Fig. 1). Although the usage of the BAK-included dorzolamide/timolol did not show the statistical significance in Cox’s hazard regression model, the patients were more likely to have PD after using dorzolamide/timolol eye drops with BAK rather than without BAK (log-rank test, p = 0.0014) when the percentage of patients with PD divided by whether the BAK was included or not is plotted as a function of time using the Kaplan–Meier survival graph (Fig. 2). This finding is supported by the prevalence of PD according to each dosing duration (< 1 year, 1–3 years, and > 3 years, Fig. 3). The prevalence of PD in the BAK(+) group was significantly higher than that in the BAK(−) group when the dosing duration was over one year (9.7% vs. 2.5% in 1–3 years, and 6.3% vs. 1.6% in ≥ 3 years; p-value of 0.015, and 0.018, respectively) whereas there was no significant difference in < 1 year (3.7% vs. 1.3%; p = 0.324).

## Discussion

The presence of side effects caused by topical glaucoma medications is known as the most significant factor linked to nonadherence which could lead to treatment failure^6^. Sensitization to topical beta-blocker is a well-known side effect of glaucoma medication^3,7–9^. And rarely but possibly, contact dermatitis related to co-reactions to both timolol and levobunolol were reported in a previous study^10^. On the contrary, allergic contact dermatitis due to dorzolamide is relatively rare^11^.

The most frequent cause of eyelid dermatitis is allergic contact dermatitis^12^. However, we still have no clue on the prevalence of PD caused by certain topical agents. To our knowledge, this is the first clinical study to describe the characteristics of PD patients after using dorzolamide/timolol eye drops and the impact of BAK on the prevalence of PD in a consecutively enrolled population. We confirmed that the elimination of BAK in dorzolamide/timolol eye drops could help patients minimize the probability of having PD. This fixed combination drug has been the second most common eye drop for the treatment of glaucoma in South Korea following the latanoprost eye drops since 2013^13^. In our study, the prevalence of PD after using dorzolamide/timolol eye drops was 2.26%, which is rare; nevertheless, it should not be ignored since the occurrence of PD demands seeking other treatment options. Worse VFI and ocular surgery history were linked to high probability of having PD, probably due to higher chance and longer duration of previous chronic contact with preserved glaucoma eye drops considering chronic nature of glaucoma treatment since preservative-free glaucoma eye drops were first introduced within no more than 10 years in South Korea.

Interestingly, the mean dosing duration before the occurrence of PD in our study was relatively longer than that in a previous report from the UK (approximately 119 weeks vs. 20 weeks, respectively)^14^. We can speculate that there is an ethnic difference even though the eye drop evaluated in our study contains two potentially allergic agents, whereas the UK study had dealt with only one. In 2016, Deleo et al. reported that there were statistically different proportions of positive patch tests to certain allergens between black and white subjects in the North American Contact Dermatitis Group from 1998 to 2006 with suspected allergic contact dermatitis^15^. And the authors suggested this might also relate to culturally determined exposure patterns rather than genetic differences.

To minimize the loss of treatment choices, the change of regimen from twice daily to once daily can allow the resumption of topical medication by improving adherence. Fourteen out of 29 patients in the PD(+) group were rechallenged with preservative-free dorzolamide/timolol eye drops once daily or once every other day rather than the original regimen. Eleven of them (78.6%, 11/14; 9 out of 10 and 2 out of 4 in the preservative-free and preserved group, respectively) showed no recurrence of PD having the eye drops while using the new regimen, which implies the dose-dependency of this allergic reaction to the drug. Delaney et al. described four out of six patients who only resolved on stopping all topical drugs that included preservatives; the aforementioned patients were rechallenged with BAK, and no adverse reactions were noted^14^. However, in two out of four patients, the PD recurred within four days in alignment with the “causality of adverse events by drugs” criteria proposed by Naranjo et al.^16^. Still, we can consider the rechallenge with the preservative-free formulation once daily or once every other day regimen prior to ceasing the causative agents.

Jappe et al. reported that in 156 out of 332 clinically suspected patients with allergic contact PD, the patch test to the patients’ own topical drugs showed a positive predictive value of only 16%, although the negative predictive value was 90%^10^. Timolol was observed in 11.1% (21/189) of positive patch test results in their study. The reported prevalence of subjects with PD from previous literature shows a wide variation ranging from 3 to 21%^10,12,17^. The low positive predictive value may be due to a certain proportion of false-negative patch tests with beta-blockers containing eye drops. Thus, we believe that validation methods such as the “stop-restart test”, if possible, are more reasonable than patch tests in those cases where there are a number of other topical treatment options are present. Higher positive patch test rates of BAK in allergic contact PD were noted than in other types of dermatitis (1.9% vs. 0.9%, respectively)^18^, which can explain the higher rate of PD in the BAK-included formulation than in the not-included formulation shown in our results. Our data also showed 1.9 times higher rates of the BAK-included eye drop users in the PD(+) group than in the PD(−) group, although the positive rates were still low, but explainable by the relatively low positive patch test rates of BAK as described above.

There are several considerations regarding our study. This was a retrospective study that used medical records without any patch test results. Therefore, it can be argued whether these PD patients are linked to allergic contact dermatitis due to topical timolol or dorzolamide. However, false-negative results following patch tests are not infrequent. Some authors have therefore recommended the modified method of performing patch testing on stripped skin^19–21^. Delaney et al. reported that all six patients who underwent patch testing showed negative results. Pitfalls in the patch test of suspected dermatitis patients include the lack of standardized allergens and protocol, high false-negative results due to lower penetration of allergen into the back, and the lack of reliable information concerning the most feasible concentration for each allergen^22^. In our study, all 29 cases showed complete recovery of PD symptoms within 2 to 4 weeks after cessation of dorzolamide/timolol eye drops, which very much likely implies the formulation of the drug is, at the very least, the “probable” or “definite” cause for the occurrence of PD according to a well-recognized description by Naranjo et al.^16^.

Second, systemic hypertension history could be linked to the efficacy and side effects of the use of timolol eye drops. Since beta-blockers are one of the commonly prescribed drugs for controlling systemic hypertension, the combined use of timolol eye drops with oral beta-blockers can increase the sensitization of the orbital tissue, resulting in a higher rate of PD. We also found that a history of medication for systemic hypertension was related to a higher rate of PD, which supports this theory, although it did not reach statistical significance (p = 0.088), probably due to the retrospective design of our study. Cross-sensitivity of timolol with other beta-blockers is also reported, which backs this point as well^10^.

Lastly, ocular operation history was clearly linked to the prevalence of PD in our subjects. Out of 23 PD patients, four who had been using the eye drops for both eyes demonstrated unilateral manifestation of PD signs such as lid edema and erythematous change of the periorbital skin only on the same side where the ocular surgery was performed at the beginning. However, after a few weeks, PD signs also occurred in the contralateral eyelid. We can speculate this phenomenon was shown due to the fact that the eyelids were over-sensitized by some of the particular agents used during the operation (e.g. 5% betadine for preparation or mitomycin application) followed by the spread of the inflammatory cells and cytokines into the blood^23^. Chronic exposure to water and detergents can disrupt the skin barrier, making it more vulnerable to sensitization by the allergens^24^. It is also known that the increase in epidermal Langerhans cell density generated by the disruption of epidermal permeability is followed by an enhanced reaction in allergic contact dermatitis (Langerhans cell density cells/mm: 2.08 vs 5.09, control vs barrier disruption + allergen, respectively). In the same vein, chronic exposure to BAK could lead to PD occurrence, which backs our result that shows the link between BAK and PD^5^.

In conclusion, dermatitis in the periorbital area related to the use of dorzolamide/timolol eye drops is rare but significant enough to impact the course of the treatment regimen for glaucoma. It can be mitigated by choosing a preservative-free dorzolamide/timolol, especially for those with a history of ocular surgery or who have worse VFI at the beginning of topical treatment.

## Methods

**Figure 4:**
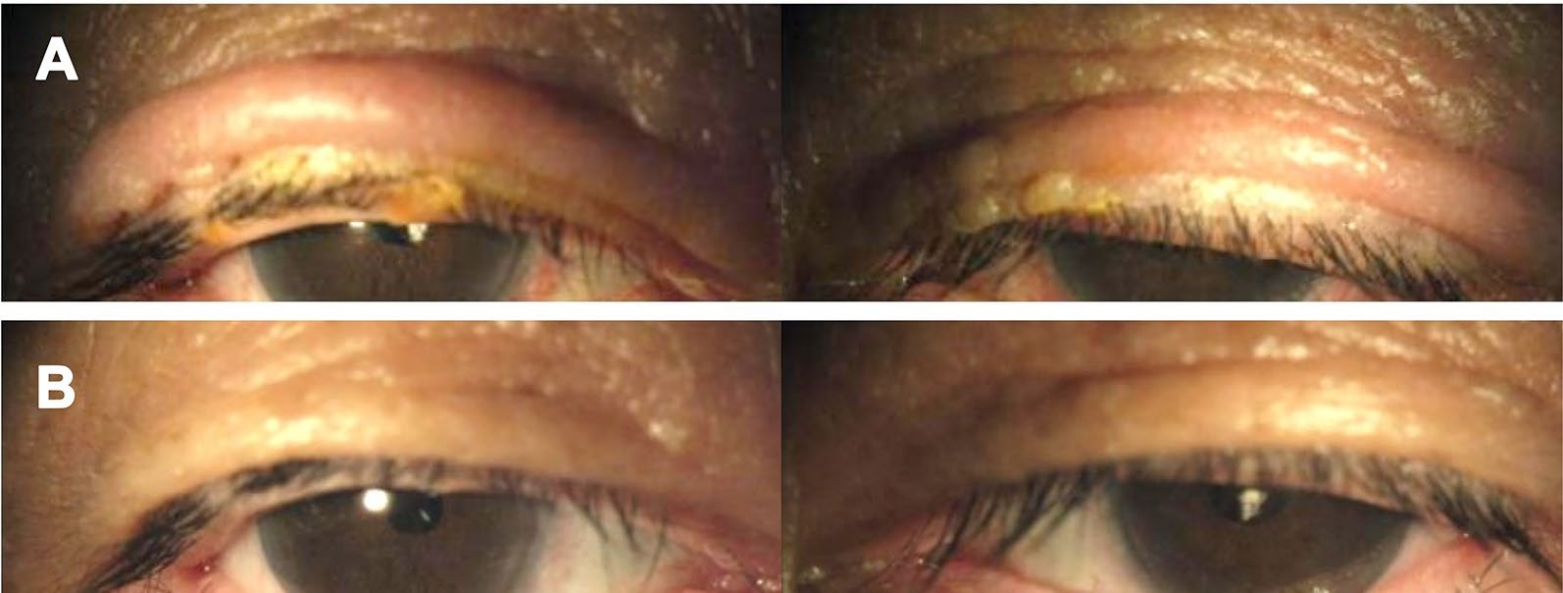
The signs of periorbital dermatitis. (**A**) Bilateral eczematous periorbital swelling after using the preservative-free dorzolamide/timolol eye drops, (**B**) improved swelling 3 weeks after cessation of the eye drops.

In this retrospective chart review, the electronic medical records of all patients who were prescribed dorzolamide/timolol eye drops with BAK or without BAK (Cosopt or Cosopt-s, respectively, Santen Pharmaceutical Co., Ltd., Japan) at CHA Bundang Medical Center between April 2012 and July 2020 were reviewed. The study was approved by the Institutional Review Board (IRB) of CHA Bundang Medical Center and was conducted at the CHA Glaucoma Clinic of CHA Bundang Medical Center in accordance with the tenets of the Declaration of Helsinki. Written informed consent was obtained from all subjects for the collection of their information without the waiver. The IRB of CHA Bundang Medical Center also approved the application for the exemption of consent for publication considering minimal harm or indiscernibility for all participants. Patients who had experienced dermatitis exclusively in the periorbital area while using dorzolamide/timolol eye drops (single fixed combination therapy) were all included in the PD(+) group, whereas the PD(−) group only included patients who had used dorzolamide/timolol eye drops for more than 6 months without experiencing PD during the whole follow-up period (Information of over-the-counter eye drops such as artificial tears was not analyzed). According to a previous study, the mean dosing duration by the time of experiencing PD was 20.4 weeks, so we thought that 6 months minimal observation duration was appropriate^14^. PD was defined as eczema at the periorbital area while using dorzolamide/timolol eye drops confirmed by an ophthalmologist during their visit based on examination of the skin and medical history review (Fig. 4). In general, we take photographs of almost all patients at every visit to keep records of their ocular surface and lid status. This helps to keep the accuracy of comparison between each visit for recognition of the change. For univariate and multivariate analyses of the risk factors of PD occurrence, we have included 10 times more subjects (n = 293) with fully accessible medical records as controls in the PD(−) group than in the PD(+) group (n = 29). Demographic features of the patients were collected from both the PD(−) and PD(+) groups.

### Statistical analysis

The demographics were compared using the chi-square test for categorical variables and independent t-tests for continuous variables. Incidence rates of PD were calculated as the number of events divided by person-time. Kaplan–Meier graphs were generated to compare the probability of remaining free of the event at each time point. Independent t-tests were used to compare the rate of PD incidence at each time period (< 1 year, 1–3 years, and > 3 years). Cox proportional hazard regression tests were used to calculate hazard rate ratios (HRRs) and associated the 95% confidence intervals (CIs). The proportional hazard assumption was confirmed using log–log plots. The risk of PD occurrence after adjusting for potential confounders (age and sex) was determined using adjusted regression models. All analyses were performed using PASW software (version 18.0; SPSS, Inc., Chicago, IL, USA). Statistical significance was set at *p* values < 0.05.
